# Expression of the gamma 2 chain of laminin-332 in eutopic and ectopic endometrium of patients with endometriosis

**DOI:** 10.1186/1477-7827-11-94

**Published:** 2013-09-26

**Authors:** Rosella Locci, Michelle Nisolle, Stefano Angioni, Jean-Michel Foidart, Carine Munaut

**Affiliations:** 1 Laboratory of Tumor and Development Biology, GIGA-Cancer, University of Liège, Tour de Pathologie (B23), Sart Tilman, B-4000 Liège, Belgium; 2 Department of Obstetrics and Gynecology, University of Liège, Hôpital la Citadelle, B-4000 Liège, Belgium; 3 Department of Surgical Sciences, Division of Obstetrics and Gynaecology, University of Cagliari, Cagliari, Italy

**Keywords:** Endometrium, Endometriosis, Cell invasion, Adhesion

## Abstract

**Background:**

Endometrial cells, which are shed by retrograde menstruation, may aberrantly express molecules involved in invasion and migration, leading to endometriosis. The aim of this study was to investigate the expression of the laminin gamma 2 chain (LAMC2) in the tissues of women with and without endometriosis.

**Methods:**

Endometrial biopsy specimens were collected from healthy volunteers and from endometriosis patients. Biopsy specimens from the corresponding endometriotic lesions were also collected. The expression of laminin gamma 2 chain was evaluated by immunohistochemistry and reverse transcription polymerase chain reaction (RT-PCR).

**Results:**

Endometrial tissue from women with or without endometriosis showed constitutive expression of LAMC2 mRNA throughout the menstrual cycle. A higher mRNA level was observed in ectopic endometrium (Ec) from women with endometriosis compared with eutopic endometrium (Eu) from women with endometriosis. Immunohistochemistry revealed a varied pattern of laminin gamma 2 chain expression, with increased epithelial expression in eutopic endometrium from women with endometriosis compared with those without endometriosis.

**Conclusions:**

The altered expression of laminin gamma 2 chain in eutopic endometrium from women with endometriosis may provide new opportunities for diagnosis and treatment.

## Background

Endometriosis is a common benign, hormone-dependent gynaecological disease that is characterised by the presence and growth of endometrial tissue outside the uterus. The most widely accepted theory is that retrograde menstruation through the fallopian tube leads to the transfer of endometrial cells into the peritoneal cavity, where they become embedded in the pelvic structures [1] (for a review, see [2]). However, this theory does not explain why more than 80% of women of reproductive age experience retrograde menstrual bleeding but do not develop endometriosis. Currently, a combination of many theories, including immunological defects, genetic predisposition and epigenetic modifications, could provide possible explanations as regarding the cause of the disease. Nevertheless, the adhesion, invasion and proliferation of ectopic endometriotic cells are all necessary for the establishment of endometriotic lesions. Notably, endometriotic cells are histologically benign but display invasive characteristics.

Laminin-332 (LN-332, previously termed laminin-5), also referred to as kalinin, nicein and epiligrin, consists of alpha 3, beta 3 and gamma 2 chains, which represent the products of three distinct genes (LAMA3, LAMB3 and LAMC2, respectively). LN-332 is a laminin isoform that is a major adhesive component of epidermal basement membranes [3,4]. *In vitro*, LN-332 promotes the attachment, spreading, scattering and migration of non-tumorigenic epithelial cells [5,6]. LN-332 also stimulates human tumour cells to form lamellipodia, leading to enhanced cell migration and invasion [7]. Immunohistochemical studies have shown that LN-332 (or its subunits) is highly expressed in various types of human cancers. In particular, the laminin gamma 2 chain is expressed in tumour cells at the invasion front or in budding tumour cells in many types of human cancers such as adenocarcinoma of the colon, breast, pancreas and lung, squamous cell carcinoma and melanoma [8,9].

Because endometriosis is characterised by the acquisition of malignant properties, such as the ability to invade surrounding tissue and disseminate to ectopic sites, the aim of the present study was to investigate the expression of the laminin gamma 2 chain in the tissues of women with and without endometriosis.

## Methods

### Sample collection

Endometriotic lesions (Ec) were removed from women (n = 25, aged 28–50 years, mean age 35.4 ± 4.8 year*s*) undergoing laparoscopy for pain or infertility (Table 1). All the women had normal documented ovulatory cycles as well as normal endocrine parameters and did not receive hormone therapy or take oral contraception for at least 3 months before surgery. Simultaneously, eutopic endometrium was obtained from the same women (EuE+). Normal endometrial tissues (EuE-) were collected from healthy non-menopausal women (n = 27, aged 20–45 years, mean age 39.2 ± 7.9 years) with spontaneous, regular menstrual cycles (26–33 days) who were undergoing laparoscopic surgery for benign gynaecologic indications (tubal ligation, ovarian cystectomy or hysterectomy).

**Table 1:** Patient characteristics

	**IHC**		**RT-PCR**	
	**Disease-free**	**Endometriosis**	**Disease-free**	**Endometriosis**
***N° of patients***	**27**	**25**	**27**	**9**
*Proliferative phase*	12	12	12	4
*Secretory phase*	15	13	15	5
***N° of patients with one lesion***		**19**		**9**
*Deep endometriosis*		7		6
*Ovarian endometriosis*		7		3
*Peritoneal endometriosis*		5		0
***N° of patients with more than one lesion***		**6**		
*Deep endometriosis*		3		
*Ovarian endometriosis*		4		
*Peritoneal endometriosis*		3		

All endometrial biopsy samples were obtained with a Cornier Pipelle suction curette (C.C.D. International, Paris, France), which allows sampling of the functional layer of the endometrium. All samples were classified according to classical histologic criteria [10].

Patients provided informed consent, and the Institutional Review Board of the University of Liège approved the collection and use of human tissue.

### Reverse transcription-polymerase chain reaction (RT-PCR) analysis

For gene expression analysis, endometrial biopsy specimens were collected from healthy volunteers (EuE-: proliferative phase, n = 12; secretory phase, n = 15) and from endometriosis patients (EuE + and Ec, n = 9) (Table 1). After surgical resection, the tissue samples were immediately frozen in liquid nitrogen. The frozen tissues were processed as previously described [11]. The specific primers (Eurogentec, Liège, Belgium) for LAMC2 mRNA were 5′-AAAGCCACGTTGAGTCAGC-3′ (forward) and 5′-TCTTCCACCTGAAAGGACTGAT-3′ (reverse). The specific primers for 28S rRNA were 5′-GTTCACCCACTAATAGGGAACGTGA-3′ (forward) and 5′-GGATTCTGACTTAGAGGCGTTCAGT-3′ (reverse). RT-PCR was performed using 10-ng aliquots of cDNA, Taq polymerase (Takara, Shiga, Japan) and 5 pmol of each primer. The specific PCR products were resolved on 10% polyacrylamide gels (Bio-Rad) and analysed with a luminescent image analyser (LAS-4000, Fujifilm) after GelStar staining (Lonza Rockland, Inc., Rockland, ME). The LAMC2 mRNA levels were expressed as ratios of the 28S rRNA as previously reported [12].

### Immunohistochemistry

Tissue samples were fixed in 4% formalin for 4–12 hours, embedded in paraffin and cut into 4-μm sections. The sections were mounted on SuperFrost Plus glass slides (Menzel-Gläser, Braunschweig, Germany), dewaxed in xylene, rehydrated and subsequently autoclaved for 11 min at 126°C and 1.4 bar in Target Retrieval Buffer (S2031 for laminin gamma 2 chain; DakoCytomation, Glostrup, Denmark) or treated with proteinase K (S3004 for cytokeratin 7 and S1699 for Ki-67; DakoCytomation, Glostrup, Denmark). Endogenous peroxidases were blocked by treatment with 3% H_2_O_2_/H_2_O for 20 min, and non-specific binding was prevented by incubation in Universal Blocking Reagent (BioGenex, San Ramon, CA, USA) for 3 min. The sections were incubated with the following primary antibodies: laminin gamma 2 chain (Dako, M7262, diluted 1:25), cytokeratin 7 (BD Biosciences, 345779, ready-to-use) and Ki-67 (Dako, M7240, diluted 1:100). The sections were washed in PBS and subsequently incubated for 30 min with EnVision + HRP (K4001, Dako) or biotinylated goat anti-mouse antibodies (Dako E0433, diluted 1:400) followed by incubation with peroxidase-labelled streptavidin for 30 min (Dako P0397, diluted 1:500). Staining was detected with 3,3′-diaminobenzidine (DAB) chromogen. The nuclei were counterstained by incubation with haematoxylin for 2 min. The sections were mounted, examined and photographed. The negative control samples were processed by omitting the primary antibody or by incubating the sections with nonspecific IgG at the same concentration as the primary antibody. Placenta was used as a positive control.

### Immunohistochemistry scoring

Immunohistochemical staining analysis was semi-quantitative. The intensity of staining was characterised as follows: no staining (0), weak but detectable (1), strong (2) or very strong (3). The percentage of positive glands was graded as follows: no positive glands (0), less than 11% (1), 11-50% (2), 51-80% (3) or greater than 80% (4). The final score was calculated by multiplying the two scores.

### Statistical analyses

The patient groups were compared using the Kruskal-Wallis test, and significant differences were further analysed via pairwise comparisons using the Mann–Whitney test. The results are presented as medians ± quartiles (25^th^-75^th^ percentile). *P* values < 0.05 were considered statistically significant.

## Results

### Laminin gamma 2 mRNA expression

**Figure 1:**
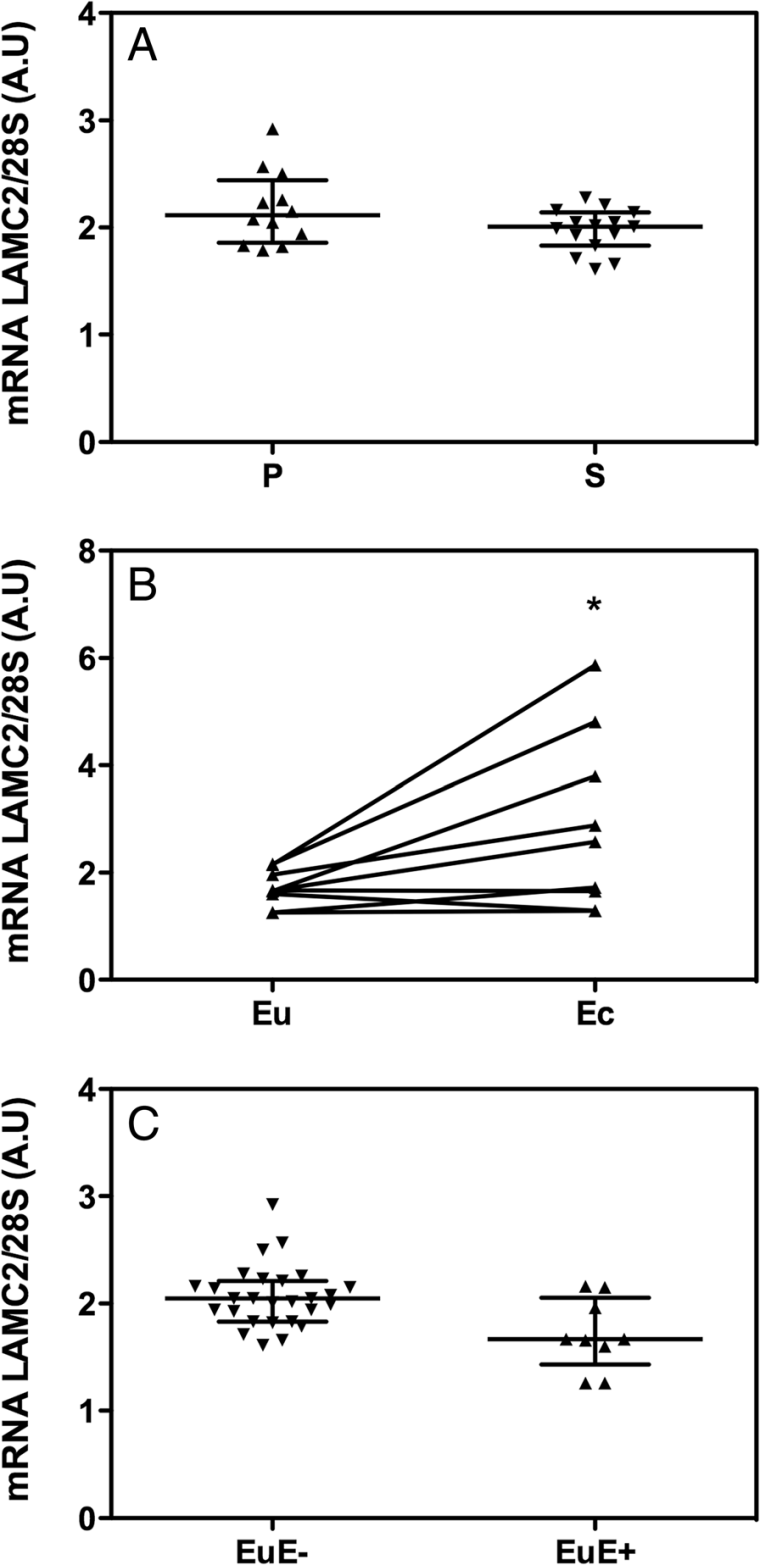
**RT-PCR analysis of LAMC2 mRNA expression.** LAMC2 mRNA levels in normal endometrium **(A)**. Paired LAMC2 mRNA expression in eutopic (Eu) and ectopic (Ec) endometrium from women with endometriosis **(B)**. Comparison of LAMC2 mRNA expression in eutopic endometrium from women without endometriosis (EuE-) and eutopic endometrium from women with endometriosis (EuE+) **(C)**. P and S indicate normal proliferative and secretory endometrium, respectively. * *P <* 0.05.

LAMC2 mRNA was detectable in the endometrium of women without endometriosis, and no differences were observed between the proliferative and secretory phases of the menstrual cycle (Figure 1A). When RT-PCR was performed in paired samples of ectopic and eutopic endometrium of women with endometriosis, an up to 3 fold increase of LAMC2 mRNA levels was detected in the ectopic endometrium (Figure 1B, *P* < 0.05). Similar LAMC2 mRNA levels were observed in the eutopic endometrium of women with and without endometriosis (Figure 1C).

### Laminin gamma 2 immunoreactivity in ectopic endometrium

**Figure 2:**
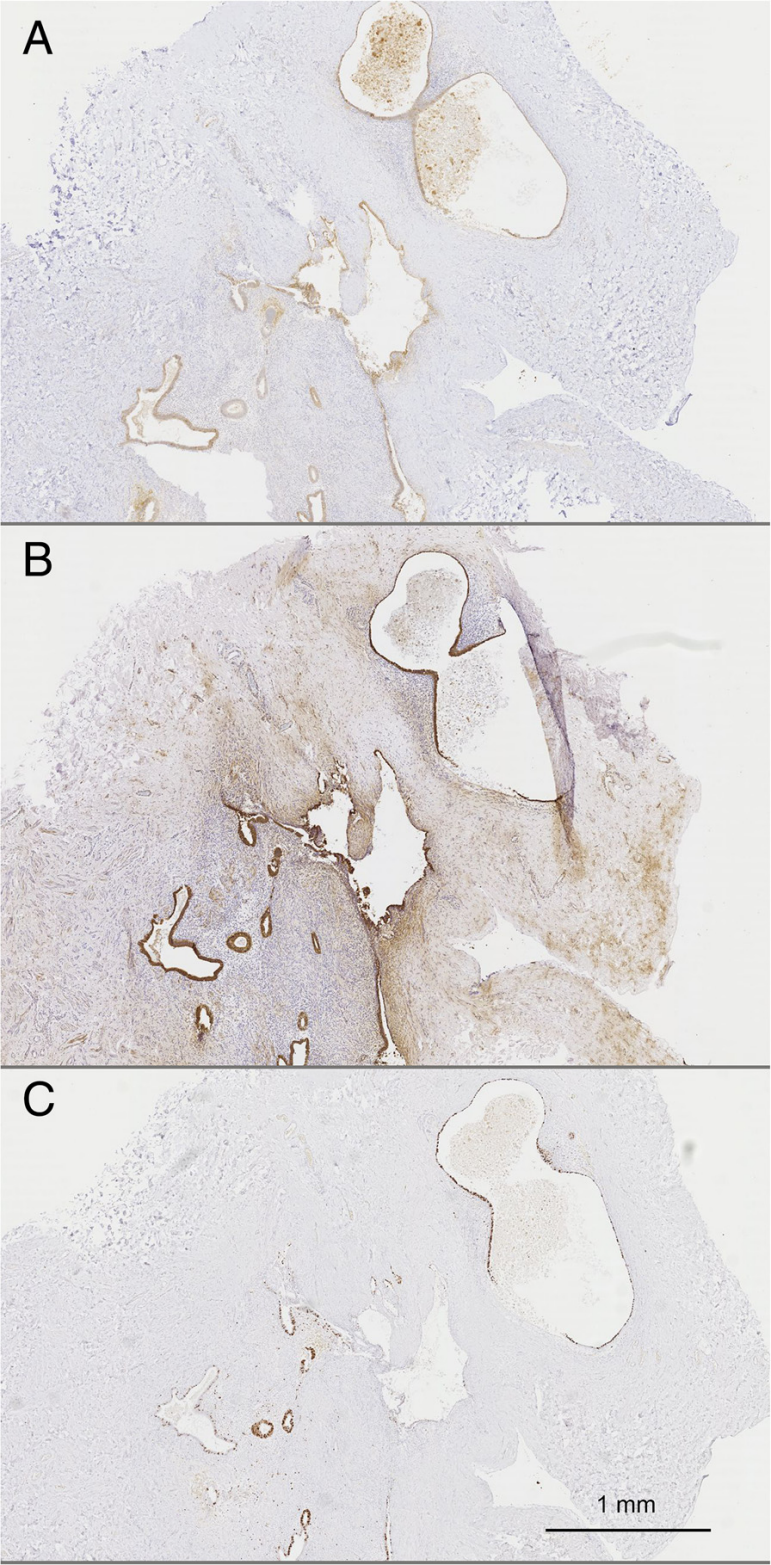
**Representative immunohistochemistry images.** For the laminin gamma 2 chain **(A)**, cytokeratin 7 (CK7, **B**) and Ki-67 **(C)** in serial sections of endometriosis lesions. Scale bar = 1 mm.

**Figure 3:**
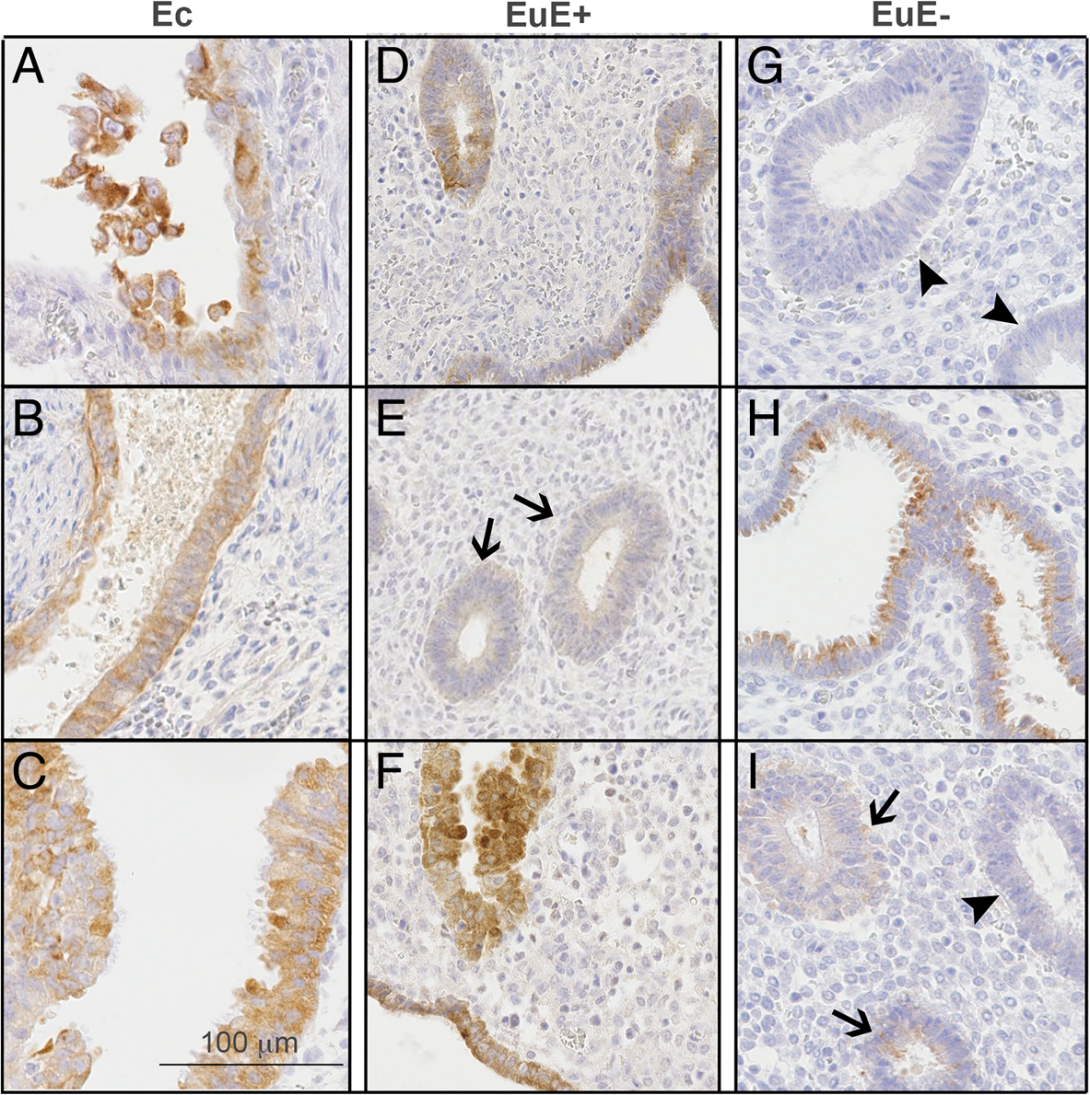
**Representative immunohistochemistry images for laminin gamma 2 chain expression.** Sections of ectopic endometrium (Ec, **A-C**), eutopic endometrium from women with endometriosis (EuE+, **D-F**) and eutopic endometrium from women without endometriosis (EuE-, **G-I**) are shown. G illustrates a negative control. Arrows indicate weakly positive glands. Arrowheads indicate negative glands. Scale bar = 100 μm.

Laminin gamma 2 expression was first investigated in ectopic endometrium (Figure 2A). Endometriotic lesions were confirmed and localised via cytokeratin 7 immunodetection, as illustrated in Figure 2B. To assess the proliferative status of the endometrial glands, Ki-67 immunoreactivity was also examined in serial sections, as illustrated in Figure 2C. Staining with antibody against laminin gamma 2 revealed a cytoplasmic expression pattern exclusively localised in epithelial cells. In endometriotic lesions, at a higher magnification, laminin gamma 2 also appeared in the cytoplasm of epithelial cells, either distributed along the basement membrane or with a more uneven distribution as illustrated in Figure 3A-C. In areas of desquamation, the epithelial cells of the dispersing glands displayed a highly intensified intracytoplasmic immunoreactivity (Figure 3A), whereas laminin gamma 2 was detected in a more linear pattern along the basement membranes of intact glands (Figure 3B). In pluristratified glands, laminin gamma 2 chain immunoreactivity was more irregular (Figure 3C).

### Laminin gamma 2 immunoreactivity in eutopic endometrium from women with and without endometriosis

Positive staining for the laminin gamma 2 chain was observed in epithelial basement membranes around individual glands and in the basement membranes underlying the endometrial surface epithelium (Figure 3D-F) in the eutopic endometrium of women with endometriosis. In normal eutopic endometrium, a similar cytoplasmic expression pattern was observed in the glandular epithelium; however, in a few cases, stronger expression was observed in the apical region of the epithelium (Figure 3G-I). Laminin gamma 2 was not observed in the stromal cells. There was no significant variation in immunoreactivity between the different menstrual phases.

### Semi-quantitative evaluation of laminin gamma 2

**Figure 4:**
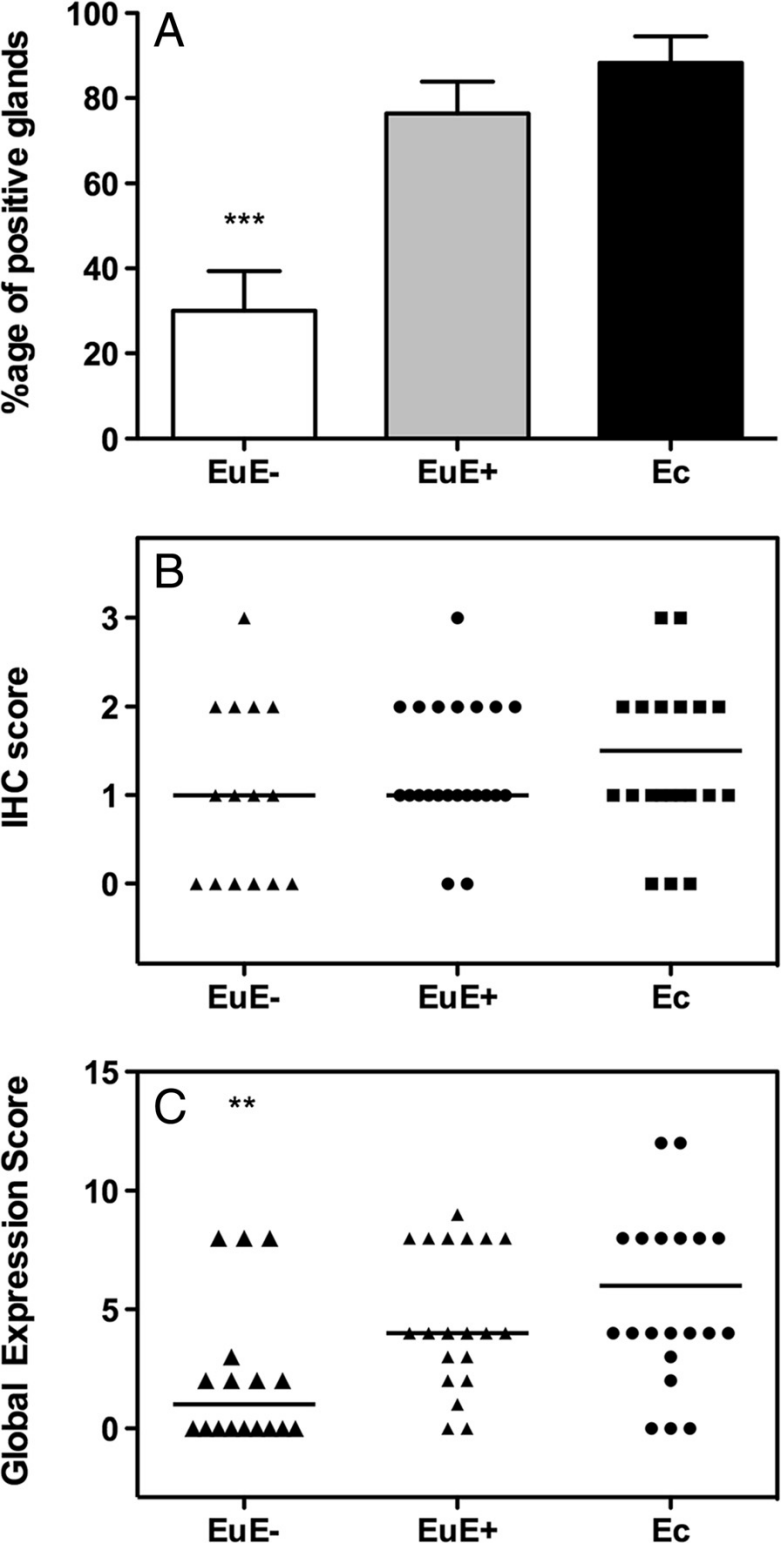
**Quantification of laminin gamma 2 chain expression in glands. (A)**, evaluation of the intensity of expression **(B)** and the global score **(C)**. Ec indicates ectopic endometrium, EuE + indicates eutopic endometrium from women with endometriosis, and EuE- indicates eutopic endometrium from women without endometriosis. ** *P <* 0.001.

To compare laminin gamma 2 expression in the ectopic and eutopic endometrium, we evaluated the percentage of positive glands in each tissue (Figure 4A). No differences were noted between eutopic and ectopic endometria from patients with endometriosis (76.4% ± 7.5% versus 88.3% ± 6.2%); however, in patients without endometriosis, the endometrium displayed the lowest percentage of laminin gamma 2 chain-expressing glands (30.1% ± 9.3%, *P* < 0.001). To better compare the intensity of laminin gamma 2 chain expression in different tissues, a semi-quantitative scoring method was performed using whole tissue sections as described above (see Methods). When we evaluated only the intensity of laminin gamma 2 staining in positive glands from the different tissues, no differences between the groups were observed; however, eutopic endometrium from women without endometriosis exhibited a null score more frequently than eutopic and ectopic endometrium from patients with endometriosis (Figures 3G and 4B). In other words, when glands were positive in EuE-, their intensity was lower than that of positive glands in EuE+. When a global scoring method was applied that considered both the number of positive glands and their intensity, no differences were observed between ectopic and eutopic endometrium in patients with endometriosis. However, endometrium from patients without endometriosis displayed a reduced global expression score compared either with eutopic endometrium from patients with endometriosis; either with ectopic endometrium (Figure 4C, *P* < 0.005).

## Discussion

In this study, LAMC2 mRNA was found to be differentially expressed in the ectopic endometrium of women with endometriosis compared with their eutopic endometrium (Figure 1B). The role of the laminin gamma 2 chain in the pathogenesis of endometriosis has not been previously evaluated, although some studies have implicated this protein in cancer invasion and metastasis. Therefore, we hypothesised that laminin gamma 2 chain could also play a role in the adhesion, migration and invasion of endometrial cells, which are required for the development of endometriosis [13]. Laminin was previously found to be expressed in the glands and stroma of eutopic and ectopic endometrium however, the type of laminin investigated was unclear [14,15]. Recently, altered expression of the LAMC1 gene was described in the endometrium of patients with endometriosis (compared with healthy endometrium) [16]. The specific expression of the laminin gamma 2 chain has not been evaluated in human endometriosis; however, the laminin gamma 2 chain was recently described as being strongly associated with the initiation of endometriosis in a mouse model [17].

Endometriosis is a benign disease, although cells from endometriotic tissue and cancer cells share the ability to spread into and invade adjacent tissue. The molecular mechanisms that drive endometriosis cells to target other tissues are largely unknown. Our present data suggest that the laminin gamma 2 chain could be involved in the invasive activity of endometriotic cells as it has been found in the majority of ectopic endometrial glands.

The comparison of laminin gamma 2 chain expression in eutopic endometrium from patients without or with endometriosis also revealed significant differences (Figure 4C). The global expression score was significantly lower in eutopic endometrium from patients without endometriosis due to the number of positive glands and their intensity. Indeed, the eutopic endometrium of women without endometriosis more often displayed weaker glandular expression of the laminin gamma 2 chain. However, when analysing LAMC2 mRNA levels, we did not observe differences between eutopic endometrium from women with and without endometriosis (Figure 1C). One possible explanation for this result could be that the mRNA analysis was performed only on a limited number of RNA samples from the eutopic endometrium of women suffering from endometriosis (n = 9), whereas for protein analysis, a greater number of patients were analysed (n = 25). The differences between gene and protein expression could also be explained by post-transcriptional modification of mRNA and mature protein as well as protein degradation [18,19]. Alternatively, small non-coding RNAs such as miRNAs, which are essentially translational repressors, could be involved in this process [20,21]. Their absence in some physiological or pathogenic conditions can contribute to increases in the amount of protein translated from a given target mRNA without altering the amount of RNA.

The facts that the laminin gamma 2 chain was expressed unevenly and that its expression was interrupted at some points could facilitate epithelial cell motility. Our results are in agreement with a previous study showing that the laminin gamma 2 chain and the alpha 3 beta 1 integrin receptor could be involved in the mechanism of endometriosis [22].

Numerous studies using immunohistochemistry and *in situ* hybridisation have shown that the laminin gamma 2 chain is localised at the leading edge of invading carcinomas and that its expression is positively correlated with invasiveness and patient survival [23]. However, other studies have shown that the expression of LN-332 is reduced during the progression of human carcinomas, and its expression is associated with lower invasive and metastatic activity [24,25]. This discordance can be explained by the overexpression of the laminin gamma 2 chain monomer in tumour cells, as the laminin alpha 3 and/or beta 3 chains are often decreased or impaired in these cells [26-28]. We have previously shown that the acquisition of a migratory phenotype in epithelial cells *in vitro* is associated with the overexpression of MT1-MMP, which can participate in the pericellular degradation of the laminin gamma 2 chain monomer deposited by the migratory cells themselves, thereby providing a modified substrate that promotes cell migration [29]. Differential laminin gamma 2 chain localisation and expression levels have been shown to be of prognostic value in colorectal [9], pancreatic [25] and lung adenocarcinomas [27] as well as gastric cancer [26]. The serum concentrations of laminin gamma 2 fragments are also useful for assessing the treatment results and clinical courses of patients with head and neck squamous cell carcinoma [30]. A weakness of our study is that sera from patients with and without endometriosis were not collected with the tissue samples.

## Conclusions

In conclusion, our study showed that the laminin gamma 2 chain is a normal component of the eutopic endometrium of women with and without endometriosis. The increased expression of the laminin gamma 2 chain in eutopic endometrium from women with endometriosis suggests a possible role for this protein in endometrial cell adhesion and, consequently, in the development of endometriosis. Laminin gamma 2 chain expression by normal endometrial cells during retrograde menstruation could contribute to their peritoneal anchoring.

Although the underlying mechanisms that lead to the development of endometriosis are not fully understood, our data indicate that the glandular cells in eutopic endometrium may phenotypically differ between women with endometriosis and disease-free women.

The altered expression of laminin gamma 2 chain in eutopic endometrium from women with endometriosis might provide new opportunities for diagnosis and treatment in the future.

## Competing interests

The authors declare that they have no competing interests.

## Authors’ contributions

RL performed some immunohistochemistry. MN and J-M F participated in the study design and helped to draft the manuscript. CM participated in the design and coordination of the study, performed the experiments and the data analysis and wrote the manuscript. All authors have read and approved the final manuscript.
